# Handlungsalgorithmus: Bauchpositionierung bei kritisch kranken Patienten

**DOI:** 10.1007/s00063-024-01146-8

**Published:** 2024-05-08

**Authors:** Carsten Hermes, Lars Krüger, Tobias Ochmann, Vanessa Erbes, Detlef Eggers, Anke Kany, Ricardo Klimpel, Victoria König, Marcel Ansorge, Anett Henck, Tobias Wittler, Thomas Bein, Stefan J Schaller

**Affiliations:** 1 Hochschule für Angewandte Wissenschaften, Hamburg (HAW Hamburg), Alexanderstr. 1, Hamburg, Deutschland; 2 Stabsstelle Projekt- und Wissensmanagement/Pflegeentwicklung Intensivpflege, Pflegedirektion, Herz- und Diabeteszentrum NRW, Universitätsklinikum der Ruhr-Universität Bochum, Bad Oeynhausen, Deutschland; 3 Klinik für Kardiologie, Internistische Intensivmedizin und Angiologie, Medizinische Intensivstation, Kath. Marienkrankenhaus gGmbH, Alfredstraße 9, Hamburg, Deutschland; 4 Fachweiterbildungsstätte für Intensiv- und Anästhesiepflege und Pädiatrische Intensiv- und Anästhesiepflege, Universitätsklinikum Gießen und Marburg, Standort Marburg, Marburg, Deutschland; 5 Akademie der Universitätsklinikum Mannheim GmbH, Mannheim, Deutschland; 6 Praxisanleitungskoordination, Pflegedirektion, Klinikum Kassel, Mönchebergstr. 41–43, Kassel, Deutschland; 7 Interdisziplinäre Intensivstation, Israelitisches Krankenhaus Hamburg, Orchideenstieg 14, Hamburg, Deutschland; 8 Internistische Intensivstation C61, Klinikum Kassel, Mönchebergstr. 41–43, Kassel, Deutschland; 9 Klinik für Anästhesiologie, Chirurgische Klinik, Universitätsklinikum Heidelberg, Im Neuenheimer Feld 420, Heidelberg, Deutschland; 10 Marienhospital Osnabrück, Interdisziplinäre Intensivstation K2, Niels-Stensen-Kliniken, Bischofstr. 1, Osnabrück, Deutschland; 11 Regensburg, Deutschland; 12 Klinik für Anästhesiologie und Intensivmedizin (CCM/CVK), Charité – Universitätsmedizin Berlin, Charitéplatz 1, Berlin, Deutschland; 13 School for Medicine and Health, Klinik für Anästhesiologie und Intensivmedizin, Technische Universität München, Ismaninger Str. 22, München, Deutschland; 14 Studiengang „Erweiterte Klinische Pflege M.Sc und B.Sc.“, Akkon Hochschule für Humanwissenschaften, Berlin, Deutschland; 15 Friedrich-Ebert-Straße 60, 53177 Bonn, Deutschland

## Einleitung

Die komplette Bauchposition/Bauchlagerung (180°-Drehung) zeigt nachweislich positive Effekte auf die Oxygenierung, besonders beim akuten Lungenversagen („acute respiratory distress syndrome“, ARDS; [3]). Durch die Reduktion des beatmungsassoziierten Lungenschadens gibt es weitere Vorteile hinsichtlich der Mortalität, die jedoch nicht (ausschließlich) auf die Verbesserung der Oxygenierung zurückgeführt werden können [1].

**Abb. 1:**
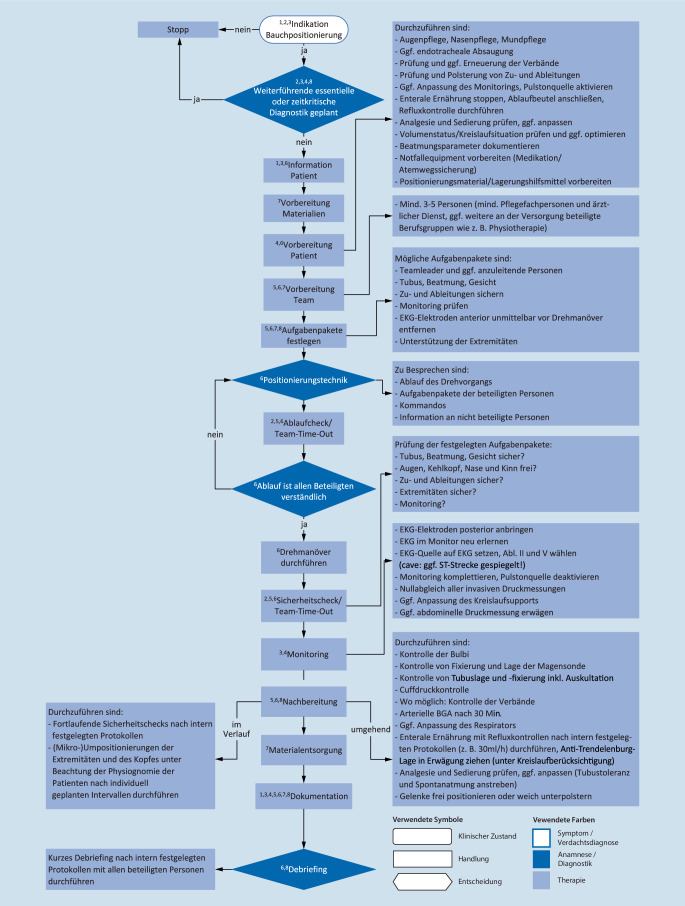
Flussdiagramm zur Bauchlagerung bei kritisch kranken Patienten

Dieser Handlungsalgorithmus (Abb. 1) bezieht sich auf die im Jahr 2023 aktualisierte S3-Leitlinie „Lagerungstherapie und Mobilisation von kritisch Erkrankten auf Intensivstationen“ [2] sowie auf die Praxisempfehlung der American Association of Critical-Care Nurses (AACN; [8]).

## ^1^Indikation Bauchlagerung bei ARDS [2, 8]

- p_a_O_2_/F_I_O_2_ < 150 mm Hg bei intubierten Patienten (trotz Optimierung der Beatmung)
- Akut hypoxisches Lungenversagen bei COVID-19 plus nichtinvasive Beatmung (NIV) → *„awake-proning“*

## ^2^Kontraindikationen [2, 8]

- Offenes Abdomen
- Wirbelsäuleninstabilität
- Erhöhter intrakranieller Druck
- Hämodynamisch relevante Herzrhythmusstörungen
- Schock

## ^3^Therapeutische Ziele [2, 8]

- Verbesserung des Ventilations-Perfusions-Verhältnis (V/Q)
- Reduktion von Atelektasen
- Verminderung des (beatmungsassoziierten) Lungenschadens
- Sekretmobilisation
- Atemarbeit ↓
- Mortalität ↓
- Vermeidung von Komplikationen

## ^4^Therapieprinzipien [2, 8]

- Dauer: mind. 12 h, ideal 16 h, unklar, ob noch länger vorteilhaft
- Auch bei vv-ECMO-Therapie möglich
- Beenden, wenn mind. 2 Versuche erfolglos oder anhaltende Verbesserung der Oxygenierung in Rückenlage (RL) erreicht ist (4 h in RL: p_a_O_2_/F_I_O_2_ ≥ 150; PEEP ≤ 10 cm H_2_O; F_I_O_2_ ≤ 0,6)
- Inkomplette Bauchlagerung (≤ 180°) therapeutisch nicht geeignet
- Lungenprotektive Beatmung

## ^5^Komplikationen [2, 8]

- Druckulzerationen und Haut‑/Schleimhautdefekte
- Augenschäden und Ödeme
- Dislokationen von Zu- und Ableitungen
- Herzrhythmusstörungen
- Kreislaufinstabilität insbesondere beim Wendemanöver oder nach Rückkehr in Rückenlage
- Reflux von Mageninhalt
- Verletzung/Reizung des Plexus brachialis

## ^6^Allgemeine Maßnahmen [2, 4, 8, 9]

- Erforderliche Ressourcen durch Klinikträger bereitstellen
- Teamleitung hat Erfahrung mit Bauchlage
- Interprofessionelle Fortbildung mindestens einmal jährlich, idealerweise mit Simulationstrainings
- Training spezifischer Methoden, z. B. der *2‑Laken-Methode* für Drehvorgang
- Algorithmen und Checklisten verwenden (Beispiel: ESM 1 *Hinweise zur korrekten Bauchlage bei ARDS*)
- Erläuterung der Maßnahme für Patienten und Angehörige
- Druckulzeragefährdete Stellen prüfen und Maßnahmen einleiten (ggf. präventiv mit mehrlagigen Schaumstoffverbänden abpolstern)
- Spezielle Augen‑, Nasen- und Mundpflege [5–7]
- Beachten der Nackenfalte in BL

## ^7^Benötigte Materialien [2, 8]

- Grundausstattung für hygienisches Arbeiten sowie spezielle pflegerische Versorgung
- Geeignetes und zugelassenes Lagerungsmaterial/Positionierungshilfsmittel
- Ggf. Weichlagerungssystem
- Cave: Keine Handtücher als Lagerungshilfsmittel verwenden (Gefahr von Dekubitus)

## ^8^Therapieplanung und Dokumentation [2, 8]

- Zieldefinition
- Situationsbezogene Besonderheiten beschreiben
- Blutgasparameter (p_a_O_2_/F_I_O_2_, p_a_CO_2_) in festgelegten Abständen erheben und dokumentieren
- Ggf. Konsultation weiterer Fachexpertise (z. B. Pneumologe)
- Strukturierte Dokumentation des Ist-Zustands vordefinierter Kriterien oder mit einem lokal erstellten Assessmentinstrument (z. B. Prüfung von Fixierungen und ggf. Unterpolsterungen (soweit einsehbar) in der Pflege- und Therapieplanung)

## Supplementary Information

Hinweise zur korrekten Bauchlage bei ARDS

Dokumentationsbogen Bauchlage
