# Supplementary material for: Treatment algorithm: prone position in critically ill patients
Source: Med Klin Intensivmed Notfmed. 2024 May 8;119(7):574–7. [Article in German] doi: 10.1007/s00063-024-01146-8 (PMC11461579; doi:10.1007/s00063-024-01146-8)
Supplement: Supplementary file 1 — Hinweise zur korrekten Bauchlage bei ARDS [file 63_2024_1146_MOESM1_ESM.pdf]

Bauchlage im ARDS

DGIIN Deutsche Gesellschaft für Internistische Intensivmedizin und Notfallmedizin

**Definition**	Bauchlage bedeutet die Drehung der PatientIn aus der Rückenlage um:<br>I. 180° → komplette Bauchlage (zu bevorzugen)<br>II. 135°-180° → inkomplette Bauchlage
**Indikation**	Akutes Lungenversagen (ARDS)<br>Oxygenierungsindex ≤ 150mmHg trotz erhöhtem PEEP<br>$\text{Horowitz} - \text{Quotient: } \frac{paO_2}{FiO_2}$<br>▪ unverzügliche Umsetzung der Bauchlage nach Indikationsstellung
**Kontraindikation**	▪ Instabile Wirbelsäule<br>▪ Schwere, operative Gesichtstraumen<br>▪ Zerebrale Läsionen (wenn keine kontinuierliche oder engmaschige Kontrolle des Hirndruckes möglich ist)<br>▪ Kreislaufrelevante Herzrhythmusstörungen<br>▪ Individuelles Abwägen bei abdominell- chirurgischen Eingriffen<br><br>*<u>Besonderheit:</u>*<br>Bei einem Risiko für einen erhöhten Hirndruck muss dieser während der Bauchlage kontinuierlich überwacht werden. Der Kopf darf dabei nicht seitlich gelagert sein.<br><br>*CAVE:* Im Einzelfall sollten Nutzen & Risiko individuell abgewogen werden<br>→ ggf. inkomplette Bauchlage in Erwägung ziehen
**Ziele**	▪ Verbesserung des pulmonalen Gasaustauschs<br>→ Veränderung des Ventilations- Perfusionsverhältnisses<br>→ Steigerung der Oxygenierung<br>▪ Verminderung / Minimierung des Lungenschadens<br>▪ Sekretmobilisation<br><br>*Merke:* PatientInnen mit gleichzeitiger Reduktion des pCO_2 in Bauchlage zeigen nach 28 Tagen eine erhöhte Überlebensrate
**Zeit und Dauer**	Bauchlagerung frühzeitig erwägen:<br>▪ mindestens 12 h, optimalerweise auf 16h erweitern<br>▪ 4h Pause, ggf. erneute Bauchlagerung für mind. 12h
**Ende**	▪ anhaltende Verbesserung der Oxygenierung in Rückenlage (4 Stunden nach Rücklagerung: PaO_2/FIO_2 ≥ 150 bei einem PEEP ≤ 10 cm H_2O und einer FiO_2 ≤ 0,6)<br>▪ nach zwei erfolglosen Lagerungsversuchen<br>▪ Individuelle Indikation in Betracht ziehen und eine Evaluation eines weiteren Bauchlageversuchs zu einem späteren Zeitpunkt vornehmen
**Komplikationen**	▪ 20-30 % Gesichtsödeme<br>▪ ca. 30% Druckulzera des Gesichts, Knie und / oder Becken<br>▪ „Nicht - Toleranz“ der Beatmung (Husten, Pressen, Beatmungskomplikationen)<br>→ Sedierung adaptieren, Relaxierung in Betracht ziehen
**Pflege**	▪ Umlagerung der Extremitäten und des Kopfes möglich<br>▪ Mikrolagerungen durchführen<br>▪ Sichere Fixierung / Abpolsterung der Zu- und Ableitungen<br>▪ Vor und nach der Bauchlage angemessene und sorgfältige Hautpflege durchführen

	▪ Gesichtsödeme lassen sich durch eine Schräglage des Bettes eindämmen → *CAVE:* Kreislaufreaktion berücksichtigen<br>▪ Endotracheales Absaugen nach Bedarf möglich<br>▪ Enterale Ernährung mit 30ml/h und engmaschigen Refluxkontrollen (4-stdl.) möglich
**Lagerungsschäden**	Plexus Brachialis: Arm nicht mehr als 90 Grad auslagern<br>Auge: Abtasten ob Bulbi freiliegen, Augen mit hautfreundlichem Pflaster abkleben, Gelkissen verwenden
**Awake- Proning**	▪ Starke Empfehlung der S3 Leitlinie für COVID-19 PatientInnen<br>▪ Keine Reduktion des Mortalitätsrisikos, jedoch eine Reduktion der Intubationsindikation<br>▪ Bauchlage bei PatientInnen im Wachzustand ist nebenwirkungsarm<br>▪ Dauer: 1h bis maximale Toleranz der PatientInnen

Checkliste Bauchlage

DGIIN
Deutsche Gesellschaft für Internistische Intensivmedizin und Notfallmedizin

Vorbereitung:

**Augenpflege**	◻ Durchführen<br>◻ Ggf. Augen mit dünnem Pflaster verschließen, um kompletten Lidschluss zu gewährleisten
**Nasenpflege**	◻ Durchführen<br>◻ Fixierung der Magensonde ggf. erneuern
**Mundpflege**	◻ Durchführen<br>◻ weiche, sichere Tubusfixierung wählen<br>◻ Ausstreichen der Mundspeicheldrüsen in Erwägung ziehen<br>◻ geschlossene Absaugung konnektieren
**Vorbeugen von Gesichtsulzerationen**	◻ AutorInnen empfehlen das Abpolstern der druckulzerationsgefährdeten Stellen mittels Schaumverbänden (Stirn, Wangenknochen, Kinn und prominent vorstehende Gelenke)
**Beatmung**	◻ Cuffdruckkontrolle & Auskultation<br>◻ auf ausreichende Länge des Beatmungsschlauches achten<br>◻ ggf. Gänsegurgel verwenden<br>◻ FiO_2 auf 1,0<br>◻ Beatmungsbeutel einsatzbereit & an Sauerstoff angeschlossen
**Verbände**	◻ Wechsel notwendig?<br>◻ Intervalle beachten
**Zu- und Ableitungen**	◻ sorgfältig fixieren & ggf. Unterpolstern / Leitungen verlängern<br>◻ Ableitungsbeutel leeren<br>◻ Leitungen kopfwärts legen<br>◻ Nicht notwendige Zugangsleitung für die Umpositionierung entfernen
**Monitoring**	◻ Alarmgrenzen Patientenbezogen einstellen<br>◻ Pulstonquelle auf SpO_2 und Lautstärke auf mind. 10% stellen<br>◻ EKG- Quelle auf Arterie oder automatisch setzen<br>◻ EKG Elektroden anterior/ventral entfernen<br>◻ Vorbereiten der Neupositionierung der Elektroden nach posterior<br>◻ Nicht relevante Monitorkabel für die Umpositionierung entfernen (griffbereit lassen)<br>◻ SpO_2 Messung auf die nach oben führende Seite legen (kontinuierliche Messung während der Drehung anstreben)
**Enterale Ernährung**	◻ Stoppen<br>◻ Magensonde via Absaugung entleeren<br>◻ Sekretablaufbeutel zur Entlastung anschließen
**Analgesie & Sedierung**	◻ RASS -4 für die Umpositionierung empfohlen<br>◻ Bolusgaben der Sedierung in 10 ml Spritzen bereithalten, zum zeitnahen titrieren<br>◻ ggf. Muskelrelaxanzien in Betracht ziehen<br>CAVE: Tubustoleranz anstreben<br>CAVE: Spontanatmung während der Bauchlage anstreben
**Notfallequipment**	◻ Noradrenalin 1mg verdünnt auf 100ml (≙ 10µg/ml)<br>◻ Noradrenalinperfusor am venösen Zugang anlaufen lassen (MAP von >65mmHg), wenn nicht bereits vorhanden

	◻ Reintubationsmaterial bereithalten (schwieriger Atemweg?) (→ siehe Intubationscheckliste)
**Lagerungsmaterialien**	◻ Kopfschale für die Bauchlage mit Spiegel in Betracht ziehen<br>◻ Gelkissen vorhanden?<br>◻ Knierolle<br>◻ Abpolsterung von Druckulzerationsgefährdeten Stellen mittels Schaumverbänden in Betracht ziehen<br>◻ Bei inkompletter Bauchlage (Bettdecke vorfalten, Stillkissen bereithalten)<br>*CAVE:* Keine Handtücher verwenden

Durchführung:

**Teamstärke**	◻ mind. 3 Pflegende<br>◻ mind. ein Arzt/ Ärztin
**Technik**	◻ Im Team vor Beginn für eine Umpositionierungstechnik entscheiden<br>◻ Bei der Zweilakentechnik Laken bereithalten
**Besprechen**	◻ Vorbereitungen durchgehen<br>◻ Jedes Teammitglied kennt seine Aufgabe<br>◻ Kommando besprechen<br>◻ Andere Teammitglieder, die nicht mit an der Umpositionierung mitwirken, informieren
**Aufgaben**	◻ Tubus / Beatmung / Gesicht?<br>◻ Zu- und Ableitungen sicher?<br>◻ Monitoring / Alarmgrenzen?<br>◻ Extremitätenlagerung (*CAVE:* Plexusschäden)?

Nachbereitung:

**Augenpflege**	◻ Bulbi kontrollieren
**Nasenpflege**	◻ Fixierung und Lage der Magensonde kontrollieren
**Mundpflege**	◻ Tubusfixierung kontrollieren
**Beatmung**	◻ Cuffdruckkontrolle<br>◻ Tubuslagekontrolle & Auskultation (Zahnreihenkontrolle möglich?)<br>◻ Arterielle BGA 30 Min. nach Umpositionierung<br>◻ FiO_2- Einstellung nach BGA adaptieren
**Verbände**	◻ kontrollieren
**Zu- und Ableitungen**	◻ Kontrollieren, frei lagern, ggf. Unterpolstern<br>◻ Zugangsleitungen komplettieren
**Monitoring**	◻ EKG Elektroden posterior anbringen<br>◻ EKG im Monitor Neuerlernen<br>◻ EKG-Quelle auf EKG setzen, Ableitungen II & V wählen (zur Beurteilung der ST-Strecke; *CAVE:* diese ist jetzt „gespiegelt“)<br>◻ Monitoring komplettieren<br>◻ Nullabgleich aller invasiven Druckmessungen<br>◻ Abdominelle Druckmessung in Betracht ziehen
**Enterale Ernährung**	◻ Bis 30ml/h<br>◻ 4 - stündliche Refluxkontrollen
**Analgesie & Sedierung**	◻ RASS –2/ -3 empfohlen<br>◻ ggf. Muskelrelaxation in Betracht ziehen (RASS-Evaluation)<br>*CAVE:* Tubustoleranz & Spontanatmung anstreben
**Lagerung**	◻ Gelenke frei lagern oder weich unterpolstern<br>◻ Mikrolagerung der Extremitäten und des Kopfes (Parotitisprophylaxe)<br>◻ Bei inkompletter Bauchlage einen Seitenwechsel nach 8h durchführen<br>◻ Anti – Trendelenburg - Lage unter Kreislaufberücksichtigung in Erwägung ziehen<br>○ Reduzierung der Gesichtsödeme<br>○ Verbesserung der Oxygenierung<br>○ Reduzierte Atemarbeit<br>○ Reduktion des Risikos einer Aspiration<br>○ Reduktion des intrazerebralen Drucks

**Dokumentation**	☐ Dokumentation der Bauchlage<br>☐ BGAs mit Berücksichtigung der Messwerte<br>☐ FiO_2 und ggf. Temperaturkorrektur beim Einpflegen der BGA eingeben, um einen Horowitz-Quotienten zu ermitteln, ggf. errechnen<br>(Formel s.o.)<br>☐ Horowitz-Quotienten in der Tageskurve notieren<br>☐ AutorInnen der Checkliste empfehlen die Erstellung eines Bauchlagerungsprotokolls zur Dokumentation von Lagerungsrhythmen und Mikrolagerungen

Verfasser:

Vanessa Erbes, B.A.
Fachgesundheits- und Krankenpflegerin für Intensivpflege und Anästhesie
Mitglied Deutsche Gesellschaft für Internistische Intensivmedizin und Notfallmedizin (DGIIN)

Tobias Ochmann
Fachgesundheits- und Krankenpfleger für Intensivpflege und Anästhesie
Stellv. Sprecher Sektion Pflege
Deutsche Gesellschaft für Internistische Intensivmedizin und Notfallmedizin (DGIIN)

Carsten Hermes
Fachgesundheits- und Krankenpfleger für Intensivpflege und Anästhesie
Sprecher Sektion Pflege
Deutsche Gesellschaft für Internistische Intensivmedizin und Notfallmedizin (DGIIN)

Download aktuellste Version

https://dgiin.de/pflege.html

Literatur:

Deutsche Gesellschaft für Anästhesiologie und Intensivmedizin e.V. (DGAI) (2023). S3-Leitlinie: „Lagerungstherapie und Mobilisation von kritisch Erkrankten auf Intensivstation https://www.awmf.org/leitlinien/detail/ll/001-015.html (letzter Aufruf: 23.03.2020)

Deutsche Gesellschaft für Anästhesiologie und Intensivmedizin (DGAI) (2017). S3-Leitlinie Invasive Beatmung und Einsatz extrakorporaler Verfahren bei akuter respiratorischer Insuffizienz unter: https://www.awmf.org/leitlinien/detail/ll/001-021.html (letzter Aufruf: 23.03.2020)

Gattinoni, L., Vagginelli, F., Carlesso, E. Taccone, P., Conte, V. Chiumello, D., Valenza, F., Caironi, P., Pesenti, A. (2004). Decrease in PaCO2 with prone position is predictive of improved outcome in acute respiratory distress syndrome. Critical care medicine. 31. 2727-33.

Hermes, C.; Müller- Wolf, T. (2016): Arbeitsplatz und Patient: von der Vorbereitung bis zur Ausleitung. In: Anästhesie und Intensivmedizin für die Fachpflege. Larsen, R. (Hrsg.). 9. Vollständige überarbeitete Auflage. Springer Verlag. Berlin Heidelberg

Hermes, C., Nydahl, P., Henzler, D., Bein, T. (2016). Lagerungstherapie und Frühmobilisation auf der Intensivstation: Erkenntnisse aus der aktuellen Leitlinie 2015. Medizinische Klinik - Intensivmedizin und Notfallmedizin.

Krüger, L. (2017): Mobilisation und Lagerung In: Klinikleitfaden Intensivpflege. Knipfer, E.; Koche, E. (Hrsg.) 6. Auflage. Urban und Fischer. München

Peko, L., Barakat- Johnson, M., Gefen, A. (2020): Protecting prone positioned patients from facial pressure ulcers using prophylactic dressings: A timely biomechanical analysis in the context of the COVID- 19 pandemic. Wiley Online Liberay. 1595- 1606

Vogt, T., Sensen, B., Kluge, S. (2019). Bauchlagerung bei Beatmung – Schritt für Schritt. DMW - Deutsche Medizinische Wochenschrift. 144. 978-981.
