# Supplementary material for: Treatment algorithm: prone position in critically ill patients
Source: Med Klin Intensivmed Notfmed. 2024 May 8;119(7):574–7. [Article in German] doi: 10.1007/s00063-024-01146-8 (PMC11461579; doi:10.1007/s00063-024-01146-8)
Supplement: Supplementary file 2 — Dokumentationsbogen Bauchlage [file 63_2024_1146_MOESM2_ESM.pdf]

DGIIN Deutsche Gesellschaft für Internistische Intensivmedizin und Notfallmedizin

Bauchlage
Dokumentationsbogen

Klinik
Station

Patientenetikett

Allgemeines zur dokumentierten Bauchlage

Laufende Nummer __________ Beginn: ____________________ (Datum, Uhrzeit)

Ende: ____________________ (Datum, Uhrzeit)

Position ☐ 180° ☐ 135° → Begründung: ___________________________

Hautdefekte bereits vorhanden ☐ ja ☐ nein (→ wenn ja, dann Beschreibung auf Seite 2)

Katecholamintherapie ☐ ja ☐ nein

Patientenvorbereitung

Augen ☐ Augensalbe ☐ Augenpflaster ☐ sonstiges ____________

Polsterung gefährdeter Hautareale ☐ Kinn ☐ Stirn ☐ sonstiges ____________

☐ mehrlagiger Schaumverband ☐ Produkt: __________

Beatmungszugang ☐ Tubus ☐ TK ☐ NIV ☐ HFNC

☐ weiches Tubusband ☐ Maske / Kanüle/ Nasenbrille gepolstert

☐ Zu- & Ableitungen gepolstert

Eingesetzte Lagerungsmaterialien

Thorax _____ Decken _____ Kissen ☐ + PUR*-Schaum Matte

Becken _____ Decken _____ Kissen ☐ + PUR-Schaum Matte

Körper ☐ spezielles Polsterset ☐ + PUR-Schaum Matte ☐ Thorax ☐ Becken ☐ Arme

☐ Spezialbett: ________________ ☐ Spezialmatratze: ________________

Kopf ☐ PUR-Schaum Kissen (Bezeichnung: __________)

☐ Kopfschale mit Schaumstoffeinsatz (Bezeichnung: __________ , Größe: ____)

Mikrolagerung Kopf / Arme / Beine

**Uhrzeit**	01:__	02:__	03:__	04:__	05:__	06:__	07:__	08:__	09:__	10:__	11:__	12:__
**#**	☐	☐	☐	☐	☐	☐	☐	☐	☐	☐	☐	☐
**Hdz.**												
**Uhrzeit**	13:__	14:__	15:__	16:__	17:__	18:__	19:__	20:__	21:__	22:__	23:__	24:__
**#**	☐	☐	☐	☐	☐	☐	☐	☐	☐	☐	☐	☐
**Hdz.**												

* PUR-Schaum = Polyurethanschaum mit nachlassender Druckspannung

# = Augen, Nase, Ohren & Kehlkopf frei gelagert

DGIIN Deutsche Gesellschaft für Internistische Intensivmedizin und Notfallmedizin

Bauchlage
Dokumentationsbogen

Klinik
Station

Patientenetikett	Datum

Beurteilung vor der Bauchlage

Hautdefekte (Größe / Lokalisation) ______________________________

____

____

____

____

____

sonstiges ______________________________

____

Beurteilung nach der Bauchlage

Hautdefekte (Größe / Lokalisation) ______________________________

____

____

____

____

____

sonstiges ______________________________

____

Dokumentation angelegt ☐ ja ☐ nein ☐ inkl. Fotodokumentation

Verfasser:

Ricardo Klimpel
Fachkrankenpfleger für Intensivpflege und Anästhesie / Praxisanleitungskoordinator Intensivpflege
Deutsche Gesellschaft für Internistische Intensivmedizin und Notfallmedizin (DGIIN)

Tobias Ochmann (M. Sc.)
Pflegeexperte APN / Fachkrankenpfleger für Intensivpflege und Anästhesie
Stellv. Sprecher Sektion Pflege
Deutsche Gesellschaft für Internistische Intensivmedizin und Notfallmedizin (DGIIN)

Carsten Hermes (M. Sc.)
Pflegewissenschaftler, APN / Fachkrankenpfleger für Intensivpflege und Anästhesie
Sprecher Sektion Pflege
Deutsche Gesellschaft für Internistische Intensivmedizin und Notfallmedizin (DGIIN)

Stand: 03/2024
